# Primitive Photosynthetic Architectures Based on Self‐Organization and Chemical Evolution of Amino Acids and Metal Ions

**DOI:** 10.1002/advs.201701001

**Published:** 2018-03-09

**Authors:** Kai Liu, Xiaokang Ren, Jianxuan Sun, Qianli Zou, Xuehai Yan

**Affiliations:** ^1^ State Key Laboratory of Biochemical Engineering Institute of Process Engineering Chinese Academy of Sciences 100190 Beijing China; ^2^ University of Chinese Academy of Sciences 100049 Beijing China; ^3^ Center for Mesoscience Institute of Process Engineering Chinese Academy of Sciences 100190 Beijing China

**Keywords:** amino acids, chemical evolution, photosynthetic architectures, primitive pigments, self‐organization

## Abstract

The emergence of light‐energy‐utilizing metabolism is likely to be a critical milestone in prebiotic chemistry and the origin of life. However, how the primitive pigment is spontaneously generated still remains unknown. Herein, a primitive pigment model based on adaptive self‐organization of amino acids (Cystine, Cys) and metal ions (zinc ion, Zn^2+^) followed by chemical evolution under hydrothermal conditions is developed. The resulting hybrid microspheres are composed of radially aligned cystine/zinc (Cys/Zn) assembly decorated with carbonate‐doped zinc sulfide (C‐ZnS) nanocrystals. The part of C‐ZnS can work as a light‐harvesting antenna to capture ultraviolet and visible light, and use it in various photochemical reactions, including hydrogen (H_2_) evolution, carbon dioxide (CO_2_) photoreduction, and reduction of nicotinamide adenine dinucleotide (NAD^+^) to nicotinamide adenine dinucleotide hydride (NADH). Additionally, guest molecules (e.g., glutamate dehydrogenase, GDH) can be encapsulated within the hierarchical Cys/Zn framework, which facilitates sustainable photoenzymatic synthesis of glutamate. This study helps deepen insight into the emergent functionality (conversion of light energy) and complexity (hierarchical architecture) from interaction and reaction of prebiotic molecules. The primitive pigment model is also promising to work as an artificial photosynthetic microreactor.

## 1 Introduction

Sunlight is the most ubiquitous and reliable energy source on earth, which fuels the origin and evolution of life.^1^ Photochemical reactions may have played an important role in abiotically generating high‐energy chemical bonds to enhance molecular complexity.^2^ Contemporary phototrophic organisms have developed pigment (e.g., chlorophyll (Chl))–protein complexes to capture light and generate reducing energy for carbon dioxide fixation.^3^ Utilization of light energy inevitably requires a pigment system to convert light into chemical energy rather than dissipating into heat. A fundamental but fascinating question is how the “first” pigment appears during the history of evolution.^4^, ^5^ Porphyrins are on the biosynthetic pathway to Chl,^6^ which is viewed to be closely related to the evolution of photosynthesis.^7^ In a pioneer work, uroporphyrinogen, the first macrocycle intermediate for Chl, was investigated as photocatalysts for H_2_ evolution under ultraviolet flux, suggesting a possible mechanism for proto‐photosynthesis on the primordial earth.^7^ Porphyrin molecules can also work as essential building blocks to form high‐level photosynthetic architectures, for example, light‐harvesting antenna^8^, ^9^, ^10^, ^11^ and photoactive lipid membrane.^12^, ^13^ The tetrapyrrole macrocycles have been detected by non‐enzymatical reaction of aminoketone and diketone or ketoester based on pyrroles formation and oligomerizations.^14^, ^15^, ^16^, ^17^ However, prebiotic synthesis of porphyrin is open to question at least in the context of robust reaction of prebiotic sources in aqueous solution to give a moderate synthesis efficiency.^14^, ^18^ Actually porphyrins have been even regarded as an ideal biomarker to search for extraterrestrial life.^19^ Therefore, experimental construction of a plausible scenario for abiogenesis of primitive photosynthetic architecture coincident with prebiotic conditions still remains a big challenge.

Volcanic hydrothermal system is envisaged as habitats for origin of life, for it embodies heat energy and is rich in various nutrients (S, C, N, P) and metals (e.g., Zn, Mn, Fe, K), which is beneficial to molecular synthesis and concentration.^20^, ^21^, ^22^ Catalytic mineral surfaces can be continually created through mineral deposition, for example, the formation of metal sulfide (ferrous sulfide, ZnS) via the combination of hydrogen sulfide with metal ions.^23^, ^24^ Various organic molecules can also be synthesized in hydrothermal conditions, including amino acids, nucleotides, and lipids.^25^ The suggested pathway for chemical evolution of organic monomer (e.g., amino acids) in hydrothermal condition is to undergo polymerization to form larger molecules.^26^, ^27^ Other fates for them have been usually ignored, for example, self‐organization or degradation, mainly due to the intuition that molecular assemblies have weak stability at high temperature and the degraded components will fade by dilution.^28^, ^29^ Nevertheless, molecular self‐assembly/organization has been viewed as a critical pathway for chemical evolution to achieve higher stages of complexity, for example, procell models capable of compartmented metabolism and molecular networks with dynamic regeneration,^30^, ^31^, ^32^ and as an efficient bottom‐up approach to fabricate hierarchical architectures with enhanced functionality.^33^, ^34^, ^35^, ^36^ Organic–inorganic hybrids also present an elegant and reciprocal strategy to integrate organic molecules to work as templates and tune the growth of inorganic components into hierarchical structures.^37^, ^38^, ^39^

**Scheme 1:**
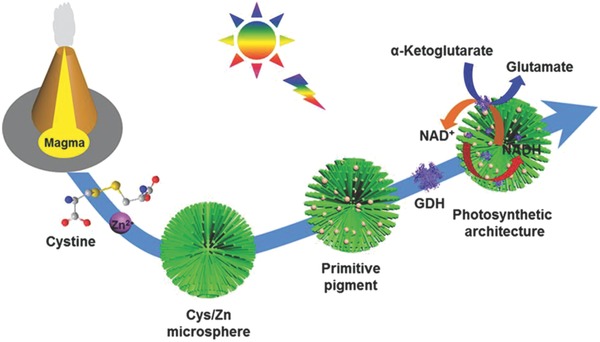
Schematic illustration of chemical evolution of cystine and zinc ion based on adaptive self‐organization in a simulated volcanic hydrothermal environment toward a primitive pigment, which can further be used as photosynthetic architecture capable of encapsulation of enzyme for photoenzymatic synthesis of glutamate.

Herein, we develop a model of primitive photosynthetic architecture capable of light harvesting and encapsulation of guest molecules based on self‐organization of amino acids and metal ions in simulated volcanic hydrothermal environment (**Scheme** **1**). Cys (oxidized form of cysteine) is chosen as a prebiotic molecular model because of its established prebiotic synthesis in hydrothermal solution and volcanic ash‐gas clouds with lightning that can be recruited to volcanic hydrothermal sites through rain‐wash.^40^, ^41^, ^42^, ^43^ Heat then essentially controls the number of nucleation sites in the assembled Cys/Zn microspheres to grow ZnS nanocrystals. Carbonate species (CO_3_^2−^) are doped on the surface of ZnS during the transformation, which makes it visible light responsive. The captured light energy can be used for H_2_ evolution, CO_2_ photoreduction, and NADH regeneration. Simultaneously, the frameworks of Cys/Zn are preserved at certain hydrothermal temperatures. The hierarchical architectures can be regarded as membrane‐free compartmentalization, where enzymes are concentrated on the surfaces of the nanorods, resulting in distinct internalized processes for photoenzymatic reactions for metabolic energy transformations (Scheme 1). The ZnS‐decorated Cys/Zn (ZnS‐Cys/Zn) microspheres generated through self‐adaptive chemical evolution provide a prebiotic conceptual scenario for origin of pigment capable of primitive photometabolism. Compared to pure ZnS mineral, a suggested prototype for prebiotic photosynthesis,^24^ the resulting ZnS‐Cys/Zn microspheres are endowed with visible light responsiveness and partitioned architecture to encapsulate guest molecules. Therefore, ZnS‐Cys/Zn microsphere can also be used as a microreactor to couple photocatalytic and enzymatic reactions.

## 2 Results and Discussion

**Figure 1:**
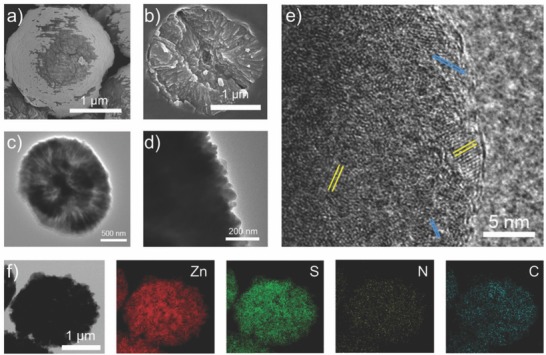
a) SEM image of a ZnS‐Cys/Zn microsphere, showing nanorods on the surface. b) SEM image of a section of a ZnS‐Cys/Zn microsphere, presenting radial nanorods from the center. c) TEM image of a ZnS‐Cys/Zn microsphere. The dark and bright regions are indicative of aligned nanorods and their space, respectively. d) Enlarged TEM image of the edge of a ZnS‐Cys/Zn microsphere. e) HRTEM image of the nanocrystallites on the edge of a ZnS‐Cys/Zn microsphere. The yellow and blue parallel lines denote the lattices of ZnS and Cys/Zn, respectively. f) HAADF‐STEM image, and elemental mapping images of a ZnS‐Cys/Zn microsphere.

The mixture of Cys and Zn^2+^ leads to form Cys/Zn microspheres via coordination‐driven self‐organization (Figure S1, Supporting Information), including hydrogen bond‐mediated stacking of coordinated molecular chains into nanorod crystals followed by their hierarchical splitting growth.^44^ The obtained Cys/Zn microspheres were placed into autoclave to simulate volcanic hydrothermal environment. After hydrothermal treatment of Cys/Zn microspheres below 160 °C (hereafter, 120 °C is taken as an example), scanning electron microscopy (SEM) images show monodispersed microspheres that are composed of nanorods (**Figure** **1**a and Figure S2, Supporting Information), suggesting that the hierarchical structure in the Cys/Zn microspheres is preserved. The nanorods are aligned to form an inherent porosity (Figure 1b–d), which may create many nanochannels for chemical entities traveling into the microspheres. High‐resolution transmission electron microscopy (HRTEM) image exhibits a lattice spacing of 0.31 nm on the surface of the nanorods (Figure 1e), which is ascribed to an interplanar distance of the (002) plane of wurtzite ZnS.^45^ The in situ formed ZnS nanocrystals have sizes within 5 nm (Figure 1e), which may facilitate photocatalytic reactions due to quantum confinement effects.^45^ The lattice fringes of Cys/Zn microspheres can be observed to be adjacent to that of ZnS (Figure 1e), indicating that the ZnS nanocrystals are deposited on the template of Cys/Zn microspheres. The high angle annular dark‐field scanning TEM (HAADF‐STEM) image and elemental mapping images suggest that the elements of Zn, S, C, and N are uniformly distributed throughout the microspheres (Figure 1f), further proving that the resulting microspheres are derived from the Cys/Zn matrices. The Cys/Zn microspheres retain structure integrity in high temperature (at least 140 °C) and only parts of Cys in the surface are decomposed to mineralize ZnS nanocrystals. This robustness in assembled structure is presumably resulted from multiple synergies of intermolecular coordination and hydrogen bonds.^46^

**Figure 2:**
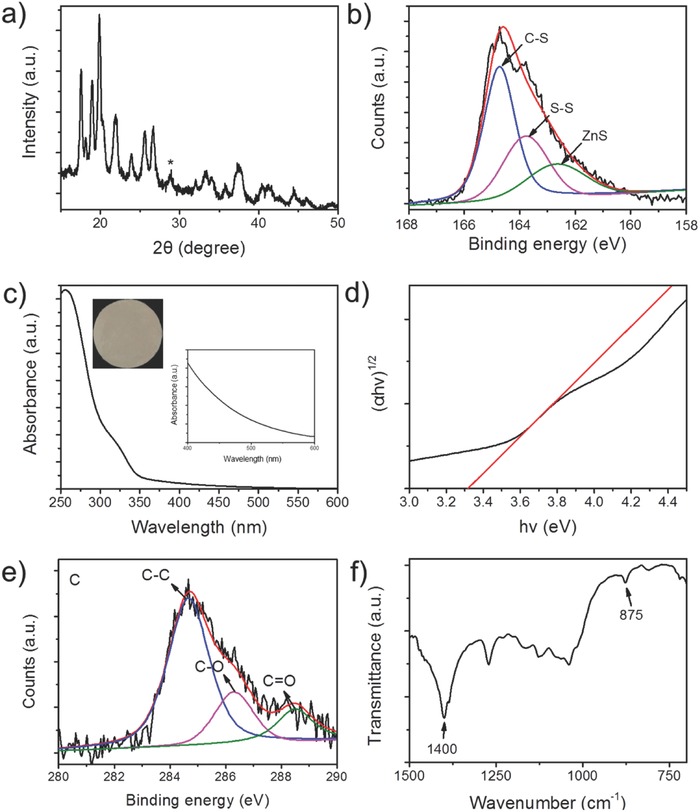
a) XRD patterns of the ZnS‐Cys/Zn microspheres. * denotes the 002 lattice plane of wurtzite ZnS. b) S 2p XPS spectra of the ZnS‐Cys/Zn microspheres. c) UV–vis diffuse reflection spectrum for the ZnS‐Cys/Zn microspheres (the insets: digital photographs of the powder of the microspheres (left) and enlarged spectrum in the range of visible light (right)). d) (*ahv*)^1/2^ versus photon energy (*hv*) of the ZnS‐Cys/Zn microspheres. e) XPS C 1s spectrum and f) FTIR spectrum of Cys/Zn after hydrothermal treatment at 200 °C for 5 h.

X‐ray powder diffraction (XRD) simultaneously shows peaks ascribed to Cys/Zn and ZnS crystals (**Figure** **2**a). In the X‐ray photoelectron spectroscopy (XPS) analysis, two states of the sulfur element belonging to organic (C—S, S—S) and inorganic sulfur (ZnS) are observed (Figure 2b). These results further confirm that the microspheres consist of Cys/Zn and ZnS components. If the hydrothermal temperature reaches 160 °C, most of the Cys/Zn component is transformed into wurtzite ZnS (Figures S2–S4, Supporting Information), indicative of heat‐driven crystallization of ZnS. Upon heating Cys/Zn gradually releases sulfur ion (S^2−^) and organic acid fragments (Figure S5, Supporting Information), due to breakage of the C—S/S—S bonds in Cys by the strong nucleophilic substitution of the oxygen atoms of water molecules.^47^ The released S^2−^ combines with Zn^2+^ to form ZnS. The Cys/Zn templates control the formation of hierarchical ZnS nanocrystals by in situ slow release of S^2−^ (Figure S6, Supporting Information), and stabilize the crystal form of ZnS presumably through coordination bonds (COO···Zn, NH_2_···Zn) (Figure S7, Supporting Information), reminiscent of the role of polyols in the controlled growth of ZnS.^48^

The Cys/Zn powders become light yellow after hydrothermal treatment, and show an obviously enhanced absorption in the visible light (400–600 nm) (Figure 2c and Figure S8, Supporting Information). The corresponding band gap is 3.32 eV (Figure 2d), which is narrower than that of pure wurtzite ZnS (3.77 eV),^49^ suggesting new electronic states. To avoid interference from Cys component, high hydrothermal treatment (200 °C) is used to make sure the complete decomposition of the Cys in the Cys/Zn, as proved by XRD analysis (Figure S3, Supporting Information). In the XPS spectra of C 1s, the peaks at 286.3 and 288 eV are observed (Figure 2e), which are ascribed to C—O and C=O bonds, respectively, arising from CO_3_^2−^.^50^ Fourier transform infrared spectroscopy (FTIR) analysis shows the peaks of 1400 and 875 cm^−1^ (Figure 2f), which can also be ascribed to CO_3_^2−^_._ 51 Decarboxylation occurs during the decomposition of many amino acids,^52^ and thus we suggest that the generated CO_2_ may convert into doped CO_3_^2−^. It has been reported that incorporation of C or N impurities can induce the visible light responsiveness of semiconductors.^53^, ^54^ Substitutional C (282.5 eV),^53^ N (397.0 eV eV) impurities,^54^ and interstitial N sites (400.0 eV)^53^ are not observed for the microspheres (Figure 2e and Figure S9, Supporting Information). Therefore, a more plausible explanation is that the CO_3_^2−^ works as an interstitial dopant.^51^ Carbonate in ZnS can generate localized electronic states located above the valence band of pure ZnS, resulting in responsiveness for visible light, a superior property for light harvesting like porphyrin.

**Figure 3:**
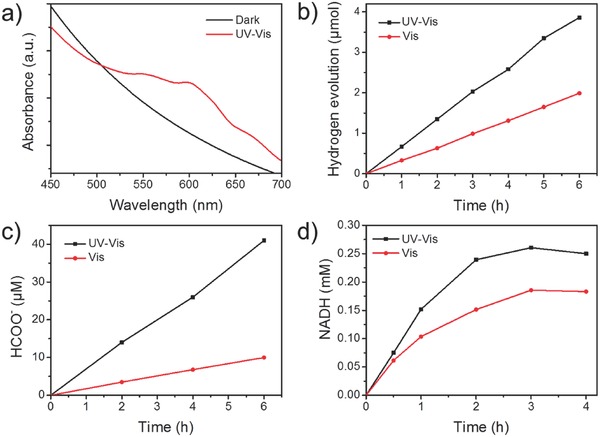
a) UV–vis spectra of the solution of ZnS‐Cys/Zn microspheres in the presence of MV^2+^ and TEOA before and after xenon lamp (UV–vis) illumination for 10 min. Time dependence of b) H_2_ evolution, c) HCOO^−^ evolution, and d) NADH regeneration on the ZnS‐Cys/Zn microspheres under UV–vis or visible light (λ ≥ 400 nm) illumination.

Proof‐of‐concept studies on the photochemical viability of ZnS‐Cys/Zn are further performed, including methyl violet (MV^2+^) photoreduction, H_2_ evolution, CO_2_ photoreduction, and NADH generation. First, ZnS‐Cys/Zn, MV^2+^, and a sacrificial electron donor (triethanolamine, TEOA) were mixed in anaerobic aqueous solutions (pH 8.0). After illumination, a growth of an absorption band at 605 nm ascribed to MV^+•^ radicals is observed (**Figure** **3**a). These results suggest that light‐driven charge separation occurs on ZnS‐Cys/Zn, and the trapped electrons in conduction band can be used to reduce MV^2+^ to MV^+•^. Therefore, the ZnS‐Cys/Zn microspheres can funnel the light energy to photochemical reduction. Biological photosynthesis uses cytochrome b_6f_ as one‐electron redox mediators/relays to sequentially deliver the electrons from the light‐harvesting complexes to the catalytic centers.^3^ Herein, the MV^2+^/^+^ couple (−0.45 vs NHE in aqueous solution) can act as a primitive electron mediator model for photocatalytic reactions.

In the presence of MV^2+^ and potassium tetrachloroplatinate (II) (K_2_PtCl_4_), H_2_ evolution is observed for the ZnS‐Cys/Zn microspheres with a rate of 900 µmol h^−1^ g^−1^ under irradiation of xenon lamp, which simulates natural sunlight (Figure 3b). Visible light (λ ≥ 400 nm) can also drive the solar‐to‐hydrogen conversion with a rate of 440 µmol h^−1^ g^−1^ (Figure 3b), which is mainly due to the excitations from the localized states. MV^+•^ radical or the excited electron in conduction band can reduce K_2_PtCl_4_ to Pt NPs, which works as electron storage for H_2_ evolution. H_2_ is not effectively generated when MV^2+^ or Pt has been omitted, especially for MV^2+^ (Figure S10, Supporting Information), suggesting that MV^2+^ is largely responsible for the photocatalytic H_2_ evolution. MV^2+^ can mediate the transfer of excited electron from ZnS‐Cys/Zn to Pt NPs, leading to enhanced charge separation and accumulation on the interface of light‐harvesting unit and reaction center. The light‐harvesting ZnS‐Cys/Zn microspheres coupled with electron mediator and reaction center can be viewed as a primitive photosystem, with features of oxidation of organic molecules by photoexcited pigment with the attendant emission of molecular H_2_.^55^ Interestingly, manganese ion can also direct the self‐assembly of Cys into microspheres, which are capable of visible light responsive H_2_ evolution after hydrothermal treatment (Figure S11, Supporting Information), suggesting that the evolutionary principle is generally suited for the primitive pigments based on adaptive self‐organization of Cys (capable of extending to S‐containing organic molecules) and metal ions (elements for semiconductive metal sulfide).

The capability of ZnS‐Cys/Zn for CO_2_ photoreduction was investigated in CO_2_‐saturated solutions containing sodium sulfide (Na_2_S). The formate (HCOO^−^) is continuously produced and reached to 41 × 10^−6^ m after 6 h illumination with a rate of 1.33 µmol h^−1^ g^−1^ (Figure 3d). The valence‐band holes can be filled by the sacrificial electron donor (Na_2_S), facilitating the transfer of conduction‐band electrons to CO_2_ to produce the radical anion CO_2_^•−^, which may be reduced via the protonation of its carbon atom to form HCOO^•^ followed by reduction to HCOO^−^.^56^ Some C2 organic acids, such as oxalate and glycolate, are also detected in the reaction system (Figure S12, Supporting Information), mainly due to dimerization of the CO_2_^•−^ radical.^57^ Visible light can drive the reduction of CO_2_ to HCOO^−^ in a lower rate (0.33 µmol h^−1^ g^−1^) (Figure 3d), indicating that the visible‐light‐excited electrons still have a sufficiently negative reduction potential. This is possibly because that the carbonate doping does not change the position of the conduction band for ZnS. Mineral surfaces (e.g., ZnS, manganese sulfide (MnS)) potentially play a central role in the photocatalytic prebiotic syntheses and conversion of building blocks for biomolecules, however, they can work only under UV irradiation.^58^, ^59^, ^60^ ZnS‐Cys/Zn “makes a breakthrough” for efficient utilization of solar energy by reciprocal self‐origination of Cys and Zn^2+^ in hydrothermal conditions.

For NADH generation, ZnS‐Cys/Zn, NAD^+^, and TEOA were mixed in Tris‐HCl buffer (10 × 10^−3^ m, pH 8.0). After illumination by a xenon lamp, the maximal NADH conversion efficiency reaches to 26% (Figure 3d and Figure S13a, Supporting Information). In addition, the generated NADH is biologically active as proved by the horseradish peroxidase (HRP) catalyzed reaction (Figure S14, Supporting Information). Proton is released from the oxidization of TEOA by the excited hole on ZnS‐Cys/Zn. Excited electrons can be directly transferred to bound NAD^+^ molecules. After receiving two electrons and one proton, NAD^+^ will convert into NADH.^61^ Under visible light irradiation, the reaction is also indeed possible, although with a lower initial reaction rate (14 vs 30 µmol h^−1^ g^−1^) and lower maximal conversion (9% vs 26%, Figure 3d and Figure S13b, Supporting Information). In photosystem I, ferredoxin and ferredoxin‐NADP‐reductase are used for nicotinamide adenine dinucleotide phosphate hydride (NADPH) regeneration.^3^ Nonenzymatic photochemical system generally requires organic electron mediator to reduce NAD^+^ to NADH.^62^ ZnS‐Cys/Zn can realize mediator‐free NADH regeneration, representing a more primitive photochemical pathway.

**Figure 4:**
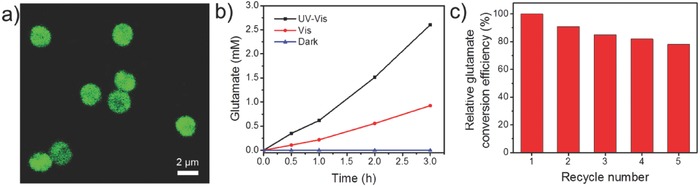
a) Confocal laser scanning microscopy (CLSM) image of ZnS‐Cys/Zn‐FITC/GDH microspheres with excitation at 488 nm and collection at 495–540 nm. b) Photoenzymatic synthesis of glutamate on ZnS‐Cys/Zn‐GDH microspheres. c) Reusability of the ZnS‐Cys/Zn‐GDH microspheres for the glutamate production.

In view of the porosity of ZnS‐Cys/Zn microspheres, we investigated their behaviors to sequester exogenous molecules. After incubation with fluorescein isothiocyanate labeled glutamate dehydrogenase (GDH) (FITC/GDH), the resulting microspheres show green emission (**Figure** **4**a, abbreviated as ZnS‐Cys/Zn‐FITC/GDH), suggesting that the enzyme molecules are incorporated into the ZnS‐Cys/Zn microspheres, probably on the surface of nanorods via coordination between carboxyl groups in enzyme and Zn^2+^ in the Cys/Zn framework.^44^ GDH (EC 1.4.1.2) is used as a model of an encapsulated molecule with a loading efficiency of 64.6%, which catalyzes the conversion of α‐ketoglutarate to glutamate ammonia assisted with ammonia and NADH. For photoenzymatic synthesis, ZnS‐Cys/Zn‐GDH microspheres, NAD^+^, α‐ketoglutarate, ammonium sulfate, and TEOA were added to Tris‐HCl buffer (10 × 10^−3^ m, pH 8.0). After illumination under simulated sunlight or visible light, glutamate is generated and the conversion yield reaches to 2.6 and 0.9 × 10^−3^ m, respectively (Figure 4b). In absence of light no glutamate is detected (Figure 4b). These results indicate that photochemical processes make NAD^+^ convert to NADH and the regenerated NADH participates in redox enzymatic reaction, which constitute a cascaded photoenzymatic reaction. After five times operation, ZnS‐Cys/Zn‐GDH remains almost 80% efficiency for glutamate production relative to the first time (Figure 4c). This is mainly because that the architectural encapsulation improves enzyme's reusability and stability, which are useful for formation of functional relays and for their integration into artificial devices.^63^ Considering the universality of the encapsulation principles, GDH can be extended to other NADH‐based reductases. Therefore, integration of ZnS‐Cys/Zn and enzymes makes up a photosynthetic reactor capable of fueling light energy to enzymatic reactions. In addition, the ZnS‐Cys/Zn microspheres can bind other guest molecule (e.g., porphyrin) via coordination and electrostatic interaction (Figure S15, Supporting Information). The resulting composites may have potential applications in dye‐sensitized solar cells^64^, ^65^ and artificial enzyme mimetics.^66^

## 3 Conclusion

In summary, a primitive photosynthetic architecture has been developed based on chemical evolution of Cys and Zn^2+^ in a volcanic hydrothermal “prebiotic soup.” The assembled Cys/Zn microspheres provide templates and precursors for heat‐driven in situ nucleation of ZnS, resulting in the decoration of ZnS nanocrystals on the nanorods from the hierarchical Cys/Zn framework. CO_3_^2−^ derived from the thermal decomposition of Cys is doped on the surface of ZnS nanocrystals, which makes it responsive to visible light. The ZnS nanocrystals on the Cys‐Zn framework have an architectural principle similar to biological light‐harvesting complex, where proteins work as a template to tune the organization of pigments. The ZnS‐Cys/Zn microspheres can further trigger various photochemical reactions, including H_2_ evolution, CO_2_ photoreduction, and NADH regeneration, reminiscent of phototrophic life. Although the prebiotic relevance of some of the molecules used in above reactions (e.g., TEOA, MV^2+^, K_2_PtCl_4_, and NAD^+^) is questionable, it is confirmed that the ZnS‐Cys/Zn microspheres can indeed induce photochemical process, including light harvesting and charge separation. We note that anaerobic H_2_ evolution has been considered as a model of proto‐photosynthesis,^7^, ^55^ and nonbiological photochemical reduction of NAD^+^ to NADH may provide an evolutionary link to biochemical reactions.^62^ Therefore, the ZnS‐Cys/Zn microspheres can be deemed as a primitive pigment model, taken together with their plausible abiogenesis. Besides CO_2_ photoreduction, ZnS‐Cys/Zn microspheres can potentially drive more strict prebiotic photochemical synthesis due to the strong reduction potential of ZnS, as shown in previous studies.^58^, ^60^ This study provides a new perspective of origin of pigment through molecular self‐organization in prebiotic conditions, which help deepen insight into the potential role of adaptive self‐organization during chemical evolution. From the view of applications, the hierarchical ZnS‐Cys/Zn microspheres are capable of guest encapsulation, which may facilitate molecular concentration and multicomponent functional coupling in confined space, for example, photoenzymatic reaction for solar energy conversation. Self‐organization and chemical evolution can not only help understand historical trajectory for origin of life but also may provide a simple but robust strategy to fabricate functional materials with emergent properties in the assistance of “natural creative force.”

## 4 Experimental Section

*Preparation of Cys/Zn Microspheres*: Cys was dissolved in ultrapure water by adjusting the pH to ≈12.0 using 1 m NaOH. Zinc chloride (ZnCl_2_) solution was obtained by dissolving ZnCl_2_ in ultrapure water. Cys/Zn microspheres were prepared by a direct mixture of Cys solution and ZnCl_2_ solution. In a typical process, 40 µL of 50 × 10^−3^ m Cys solution was added to 940 µL of ultrapure water, followed by addition of 20 µL of 100 × 10^−3^ m ZnCl_2_ aqueous solution, and the finial pH was around 8.0. After mixing, the solution became turbid immediately. White precipitates were obtained by centrifugation of the turbid solution at 8000 rpm for 10 min. The precipitates attributed to Cys/Zn microspheres were then washed by ultrapure water to remove free cystine and ZnCl_2_.

*Preparation of ZnS‐Cys/Zn Microspheres*: 20 mL of Cys/Zn aqueous solution (1 mg mL^−1^) was added into a Teflon‐lined autoclave (25 mL). The autoclave was sealed, maintained at certain temperature (120, 140, 160, or 200 °C) for 5 h, and then cooled to room temperature naturally. The products were centrifugally washed with ultrapure water for several times.

*Characterization*: SEM was carried out with S‐4800, an emission scanning electron microscope (Hitachi, Japan). TEM and HRTEM were performed on a JEM‐2100F field emission transmission electron microscope (JEOL, Japan) under 200 kV accelerating voltage. HAADF‐STEM and elemental mapping images were conducted on an FEI Tecnai G2 F20 microscope equipped on the JEM‐2100F. XRD patterns were recorded on an Empyrean diffractometer (PANalytical, Netherlands) under the following conditions: Cu Kα radiation, λ = 1.5406 Å. XPS measurements were performed on an ESCALab220i‐XL spectrometer (VG, USA). The spectra were excited using a monochromated Al KR X‐ray source 434 (1486.7 eV) operated at 15 kV. FTIR was carried out using TENSOR‐27 (Bruker, Germany). UV–vis diffuser reflectance or (absorption) spectra were recorded by a UV‐2600 spectrophotometer (Shimadzu, Japan). Thermogravimetric analysis (TGA) was performed on SDT Q600 (TA Instruments, USA) with a heating rate of 10 °C min^−1^ until heating up to 800 °C under air atmosphere. High‐resolution ESI‐MS was recorded by a Solarix 9.4T mass spectrometer (Bruker, Germany) under a spray voltage of 3 kV. CLSM images were taken by Olympus FV500 with a 60× oil immersion objective with the numerical aperture of 1.4.

*Guest Encapsulation*: Free GDH (1 mg mL^−1^) and ZnS‐Cys/Zn microspheres (5 mg mL^−1^) were mixed in 4 mL of Tris‐HCl buffer (10 × 10^−3^ m, pH 8.0). After incubation at room temperature for 12 h, the mixture was separated by centrifugation at 8000 rpm for 10 min. The amount of encapsulated GDH was calculated by the difference between total GDH content and residual GDH content in the supernatant. The concentration of GDH was determined by standard curve of GDH with known concentration based on characteristic absorbance at 280 nm. For visual observation of the distribution of GDH in the microspheres by CLSM, FITC‐GDH was used instead of GDH, but still under the same operation.

*Photocatalytic Reactions*: All photocatalytic reactions were performed in a 15 mL quartz tube equipped with a rubber stopper at room temperature and atmospheric pressure, and the stoppers were sealed with paraffin. Before illumination, all reaction systems were degassed by bubbling argon for 30 min to remove oxygen. A 350 W Xe arc lamp containing ultraviolet and visible light was used as light source. To obtain visible light, an optical filter with a cut‐off wavelength of 400 nm was equipped on the lamp. For photoreduction of MV^2+^, ZnS‐Cys/Zn (5 mg mL^−1^) MV^2+^ (2 × 10^−3^ m), and TEOA (0.1 m) were added to 4 mL of ultrapure water, and then the solution pH was adjusted to pH ≈ 8.0 by 1 m HCl. Before and after illumination, absorption spectra of the solution were measured, respectively. For H_2_ evolution, ZnS‐Cys/Zn (5 mg mL^−1^), TEOA (0.1 m), MV^2+^ (2 × 10^−3^ m), and K_2_PtCl_4_ (20 × 10^−6^ m) were added to 4 mL of ultrapure water, followed by tuning the solution pH to pH ≈ 8.0 by 1 m HCl. After illumination for certain time, 200 µL of gas was sampled from the reaction system intermittently, and analyzed by TRACE 1300 gas chromatography (Thermo Fisher Scientific, USA), using argon and 5 Å molecular sieve column as carrier gas and separating medium, respectively. For CO_2_ photoreduction, ZnS‐Cys/Zn (5 mg mL^−1^) and Na_2_S (8 × 10^−3^ m) were added to 6 mL of ultrapure water. The mixed solution was adjusted to pH 7 by bubbling CO_2_ before sealing. For HCOO^−^ analysis, ion chromatography (761 Compact, Metrohm) was used with an eluent of NaOH solution (20 × 10^−3^ m) and a flow rate of 0.85 mL min^−1^. The species in solution were identified by comparison to the chromatograms of pure chemicals. The concentrations were obtained by calibration with a series of standard solutions. For NADH regeneration, ZnS‐Cys/Zn (5 mg mL^−1^), NAD^+^ (1 × 10^−3^ m), and TEOA (0.1 m) were added to 6 mL of Tris‐HCl buffer (10 × 10^−3^ m, pH 8.0). After illumination for certain time, 400 µL of reaction solution was taken out and the concentration of NADH was evaluated by the characteristic absorbance of NADH at 340 nm, with a molar extinction coefficient of 6.22 mM^−1^ cm^−1^.

*Photoenzymatic Synthesis of Glutamate*: ZnS‐Cys/Zn‐GDH (5 mg mL^−1^), NAD^+^ (1 × 10^−3^ m), α‐ketoglutarate (5 × 10^−3^ m), ammonium sulfate (100 × 10^−3^ m), and TEOA (0.1 m) were mixed in 6 mL of Tris‐HCl buffer (10 × 10^−3^ m, pH 8.0). After illumination for certain time, 50 µL of reaction solution was taken and quantitatively estimated by using UltiMate 3000 high‐performance liquid chromatography (Thermo Fisher Scientific, USA) equipped with a C18 column (250 × 4.6 mm, 5 µm). The mobile phase was (NH_4_)_2_HPO_4_‐H_3_PO_4_ (0.5%, pH 2.5) with a flow rate of 1.0 mL min^−1^. The UV detector wavelength was set at 214 nm.

## Conflict of Interest

The authors declare no conflict of interest.

## Supporting information

Supplementary
