# Supplementary material for: Primitive Photosynthetic Architectures Based on Self‐Organization and Chemical Evolution of Amino Acids and Metal Ions
Source: Adv Sci (Weinh). 2018 Mar 9;5(6):1701001. doi: 10.1002/advs.201701001 (PMC6010005; doi:10.1002/advs.201701001)
Supplement: Supplementary file 1 — Supplementary [file ADVS-5-1701001-s001.pdf]

Copyright WILEY-VCH Verlag GmbH & Co. KGaA, 69469 Weinheim, Germany, 2018.

ADVANCED SCIENCE
Open Access

Supporting Information

for *Adv. Sci.,* DOI: 10.1002/advs.201701001

Primitive Photosynthetic Architectures Based on Self-Organization and Chemical Evolution of Amino Acids and Metal Ions

*Kai Liu, Xiaokang Ren, Jianxuan Sun, Qianli Zou, and Xuehai Yan**

Primitive Photosynthetic Architectures Based on Self-Organization and Chemical Evolution of Amino Acids and Metal Ions

*Kai Liu, Xiaokang Ren, Jianxuan Sun, Qianli Zou and Xuehai Yan**

Dr. K. Liu, X. Ren, J. Sun, Dr. Q. Zou, Prof. Dr. X. Yan
State Key Laboratory of Biochemical Engineering
Institute of Process Engineering
Chinese Academy of Sciences
100190 Beijing, China
E-mail: yanxh@ipe.ac.cn
Homepage: http://www.yan-assembly.org/
Prof. Dr. X. Yan
Center for Mesoscience
Institute of Process Engineering
Chinese Academy of Sciences
100190 Beijing, China
Dr. K. Liu, X. Ren, Prof. Dr. X. Yan
University of Chinese Academy of Sciences
100049 Beijing, China

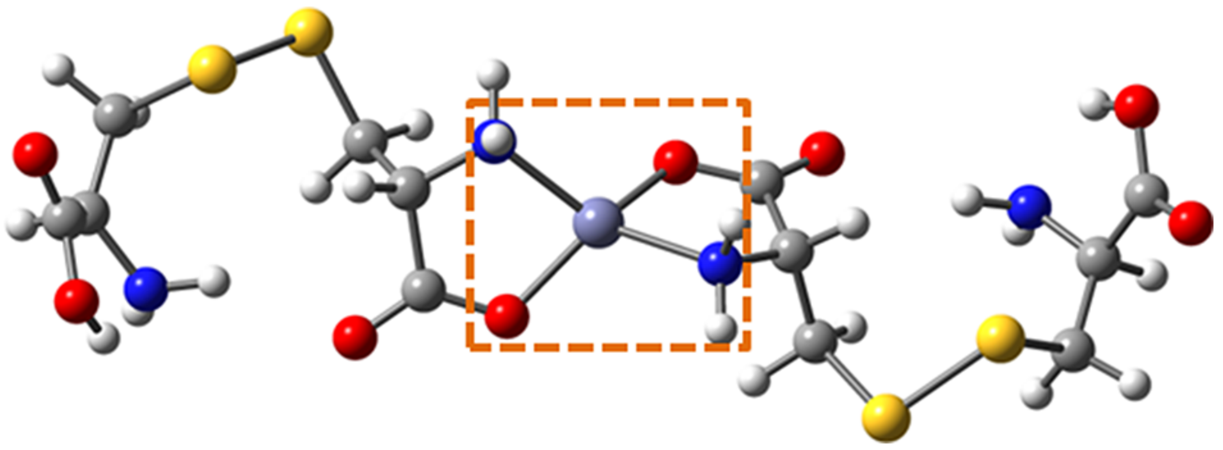

Figure S1. A suggested binding model between cystine and Zn^{2+} optimized by B3LYP/6-31G(d) in Gaussian 09. The amino and carboxyl groups in cystine are coordinated with Zn^{2+} with a coordination number of 4 (see the dashed box).

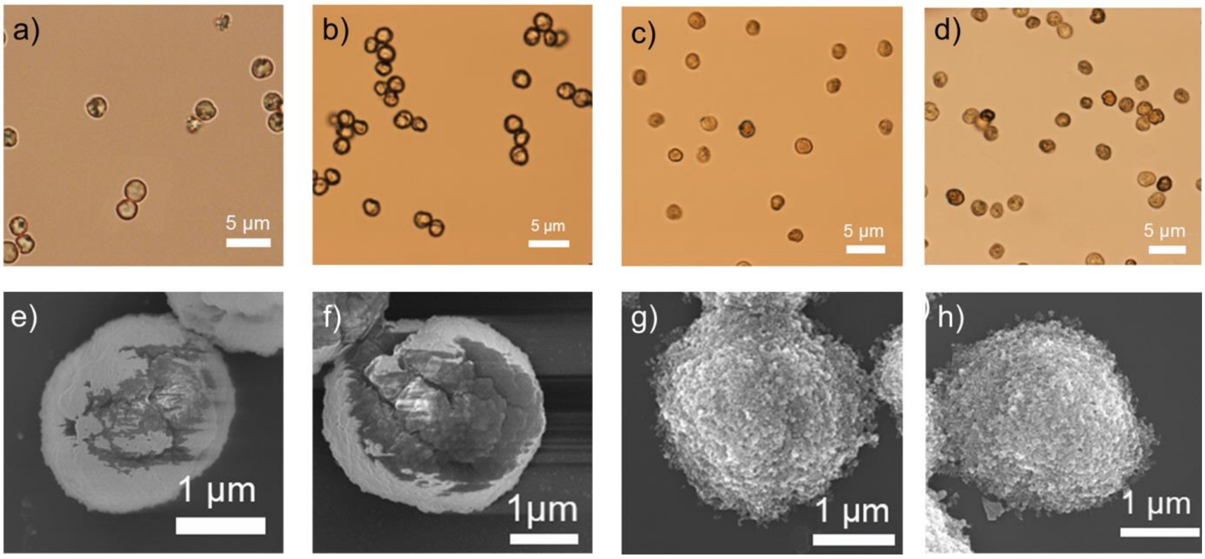

Figure S2. Optical images of the resulting microspheres a) before and b-d) after hydrothermal treatment of Cys/Zn microspheres at 140°C, 160 ^{o}C, or 200 ^{o}C for 5 h, respectively. SEM images of the resulting microspheres e) before and f-h) after hydrothermal treatment of Cys/Zn microspheres at 140°C, 160 ^{o}C, or 200 ^{o}C for 5 h, respectively. The architectural feature of Cys/Zn microspheres, which is made up of nanorods, is retained after hydrothermal treatment at 140 ^{o}C, but changes into aggregated nanoparticles at higher temperature (160°C), probably due to the enhanced thermal decomposition of the Cys component. The ZnS nuclei then grow into nanocrystallites and further aggregate into larger microspheres because of localized Ostwald-ripening.[1]

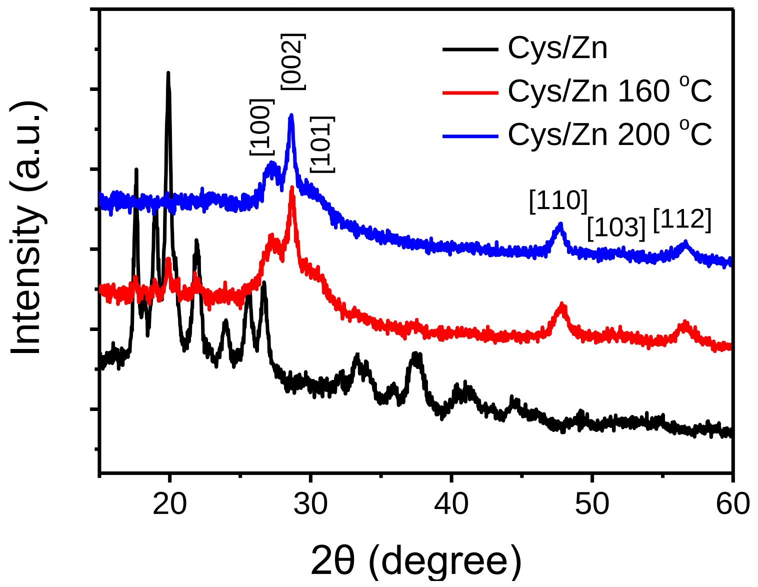

Figure S3. XRD patterns of the Cys/Zn before and after hydrothermal treatment at 160 °C and 200 °C. At 160 °C, most peaks ascribed to Cys/Zn diminish or disappear, and wurtzite ZnS becomes the main component.[2] If the hydrothermal temperature is further increased to 200 °C, only diffraction peaks of wurtzite ZnS can be observed. These results suggest that the conversion of Cys/Zn into ZnS is driven by heat.

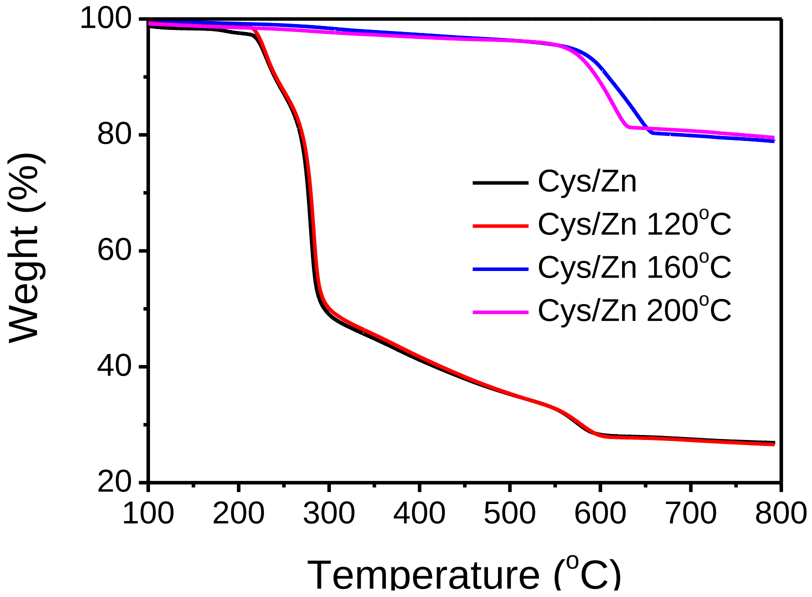

Figure S4. TGA of Cys/Zn microspheres after hydrothermal treatment at different temperature. The sharp weight loss for blank Cys/Zn and Cys/Zn microspheres treated at 120 °C before 300 °C is due to oxidation of the Cys component, the continuous weight loss between 300-600 °C possibly results from the breakage of coordination bonds. They show similar degradation curves, indicating that the component of Cys/Zn does not change significantly. The amount of ZnS embedded in the Cys/Zn microspheres is estimated to be 1.3 % based on the different weight lost at 300°C for Cys/Zn before and after hydrothermal treatment. At higher hydrothermal temperature (160 °C and 200 °C), the weight loss before 300°C almost disappears due to the previous thermal decomposition, suggesting that the obtained microspheres are mainly composed of ZnS (>97%). The weight loss between 200-500 °C is due to oxidation of residual C, N species to CO_2 and NO_2, the weight loss after 500 °C is due to oxidation of ZnS to ZnO. The content of residual C, N species is approximately 2.8 % and 2.1 % for Cys-Zn 160 °C, Cys-Zn 200 °C, respectively.

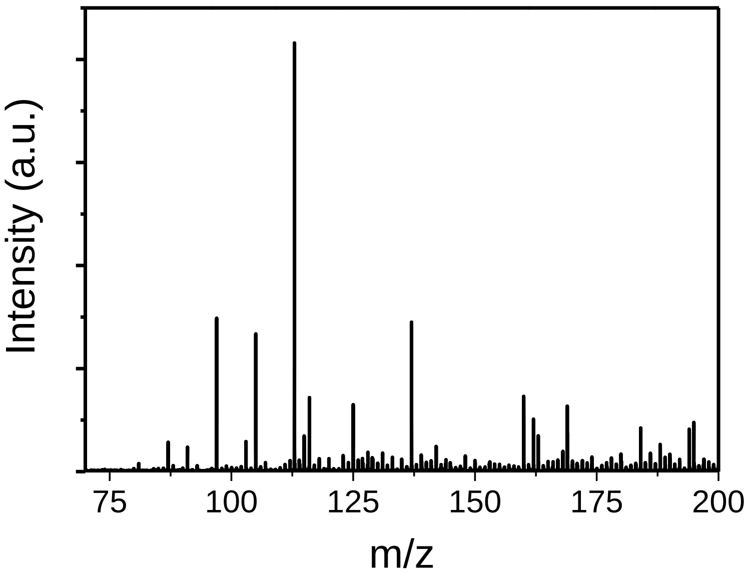

Figure S5. Electrospray Ionization Mass Spectrometry (ESI-MS) spectrum of the liquid supernatant after hydrothermal treatment of Cys/Zn at 160 ^{o}C for 5h. Some peaks are ascribed to organic acids, such as pyruvic acid ($C_3H_4O_3$, 87.0114), 2-Mercaptopropionic acid ($C_3H_6O_2S$, 105.0011), Acetic acid, 2-[(2-mercaptoethyl)amino]-2-oxo- ($C_4H_7NO_3S$, 148.0117), 5-oxothiomorpholine-3-carboxylic acid ($C_5H_7NO_3S$, 160.0094), N-acetylcysteine (C5H9NO3S, 162.0242).

a)

b)

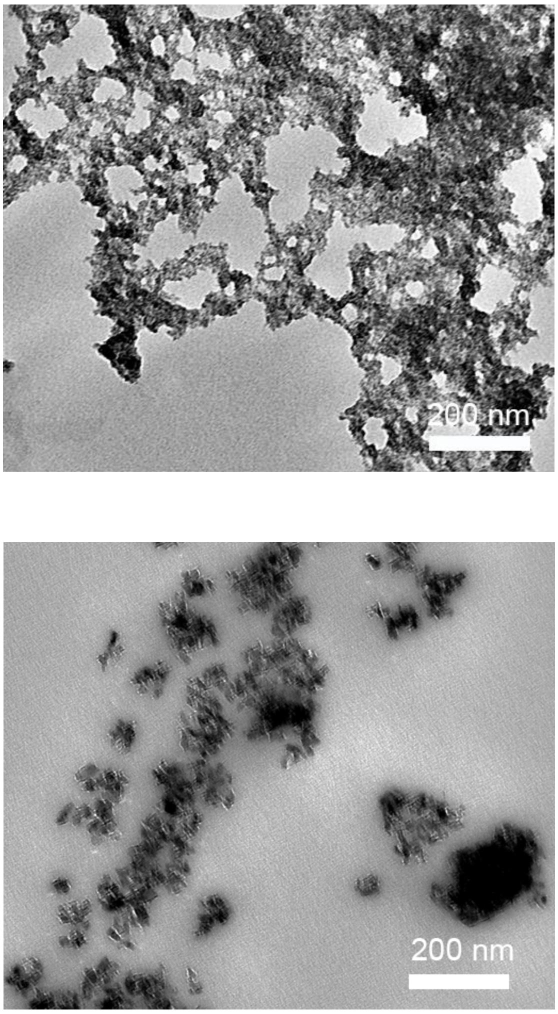

Figure S6. TEM images of ZnS nanoparticles fabricated by a direct mixture of a) Na_2S (2 mM) or b) cystine (2 mM) pre-treated at 160 ^{o}C for 5 h and $ZnCl_2$ (2 mM). The resulting nanoparticles are aggregated in a disordered way without hierarchical structures, contrary to the Cys/Zn-templated crystallization of ZnS in the ZnS-Cys/Zn microspheres.

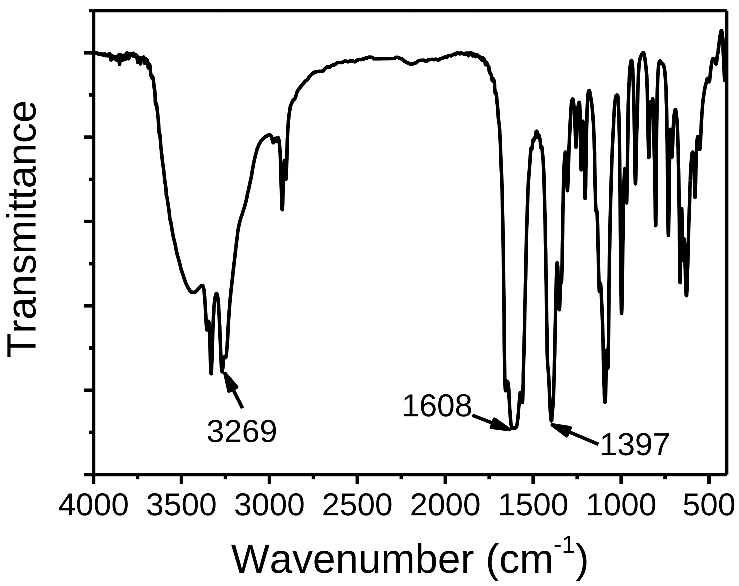

Figure S7. FTIR spectrum of the ZnS-Cys/Zn microsphere. In the spectrum COO^- symmetric stretch at 1397 cm^{-1}, asymmetric stretch at 1613 cm^{-1}, and NH_2 stretch at 3269 cm^{-1} are observed, indicative of coordination of bridging carboxyl and amino moieties of cysteine to the zinc ions.

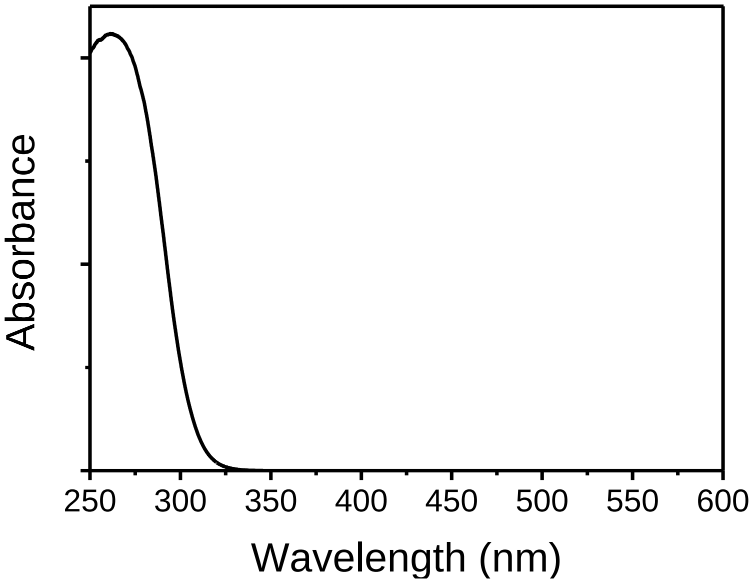

Figure S8. UV-vis diffuse reflection spectrum of the Cys/Zn powder. There is no absorbance in the visible range. The absorption peak at 260 nm is ascribed to the cystine component.

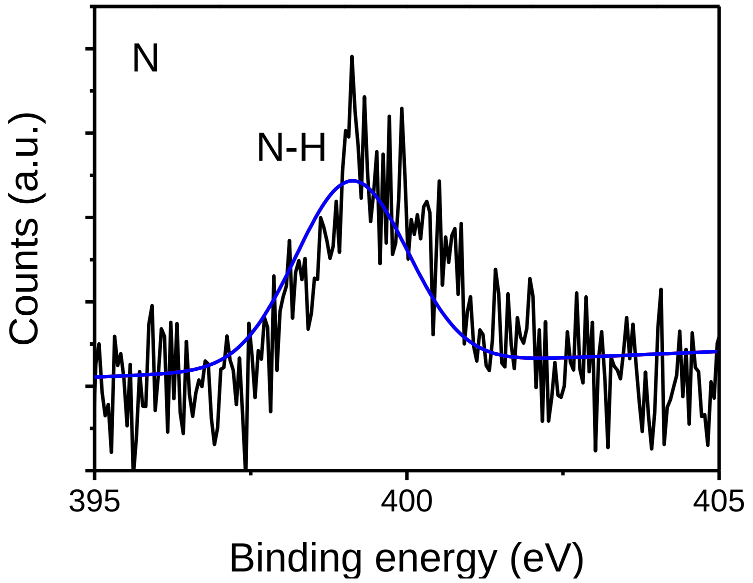

Figure S9. XPS spectrum of N 1s in Cys/Zn after hydrothermal treatment at 200 ^{o}C for 5 h. To suppress interference from cystine component, high hydrothermal treatment is used to make sure the complete decomposition of the cystine in the Cys/Zn, as proved by XRD analysis (Figure S2).

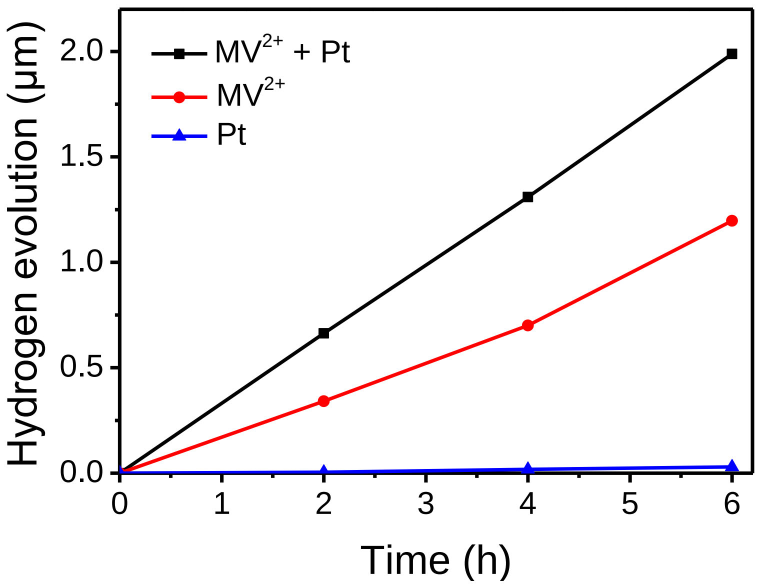

Figure S10. H_2 evolution of ZnS-Cys/Zn microspheres using TEOA (0.1 M) as sacrificial electron donor under visible light (λ≥400 nm) illumination in presence of electron mediator (MV^{2+}, 2 mM) and reaction center (Pt, 20 μM) or omitting one of them.

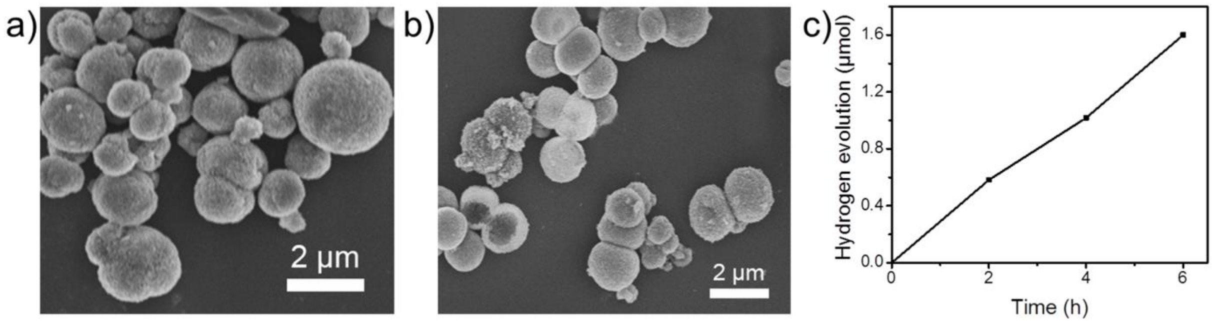

Figure S11. a) SEM image of assembled Cys/Mn microsphere after mixing cystine (2 mM) and Mn^{2+} (2 mM) in ultrapure water (pH=7). b) SEM image of the Cys/Mn microspheres after hydrothermal treatment at 120 °C for 5 hours. c) H_2 evolution of the obtained microspheres in (b) under visible light (λ≥400 nm) illumination in presence of TEOA (0.1 M), MV^{2+} (2 mM) and K_2PtCl_4 (20 μM).

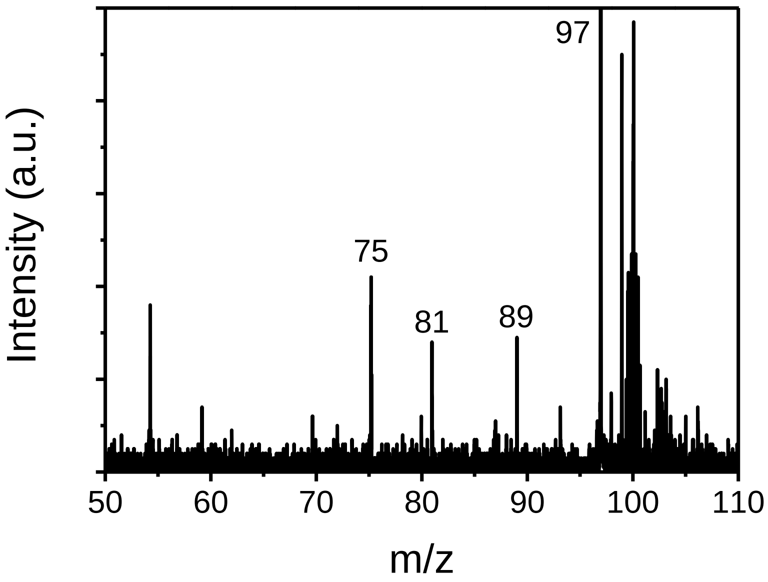

Figure S12. ESI-MS spectrum of reaction liquid of CO_2 photoreduction after illumination by a Xe lamp for 12 h, showing peaks of oxalate ($HC_2O_4^-$, m/z = 89), glycolate ($C_2H_3O_3^-$, m/z = 75), bisulfite (HSO_3^-, m/z = 81), and bisulfate (HSO_4^-, m/z = 97). HSO_3^- and HSO_4^- are generated from photocatalytic oxidation of S^{2-}.

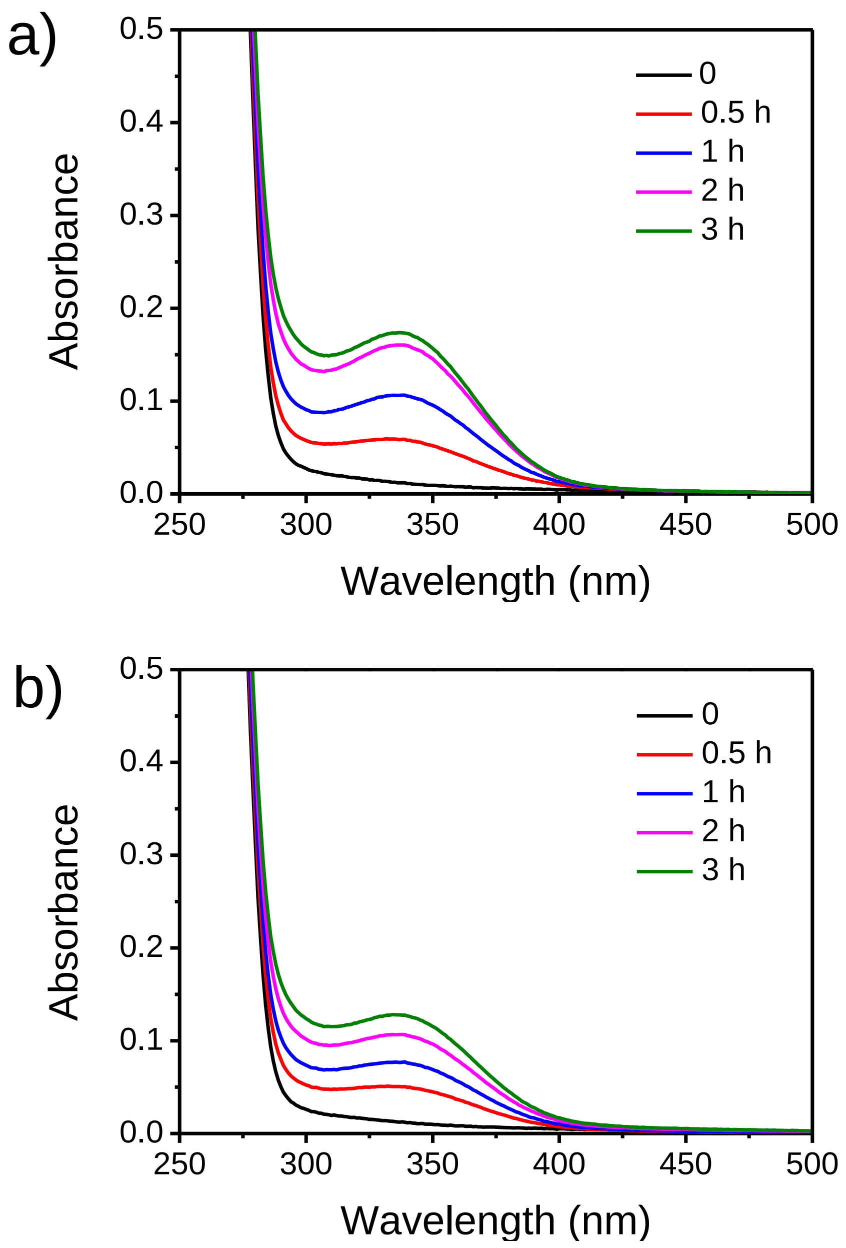

Figure S13. UV-Vis absorption spectra of NADH generated by photoreduction of NAD^+ (1 mM) under a) Xe lamp and b) visible light (λ≥400 nm) illumination.

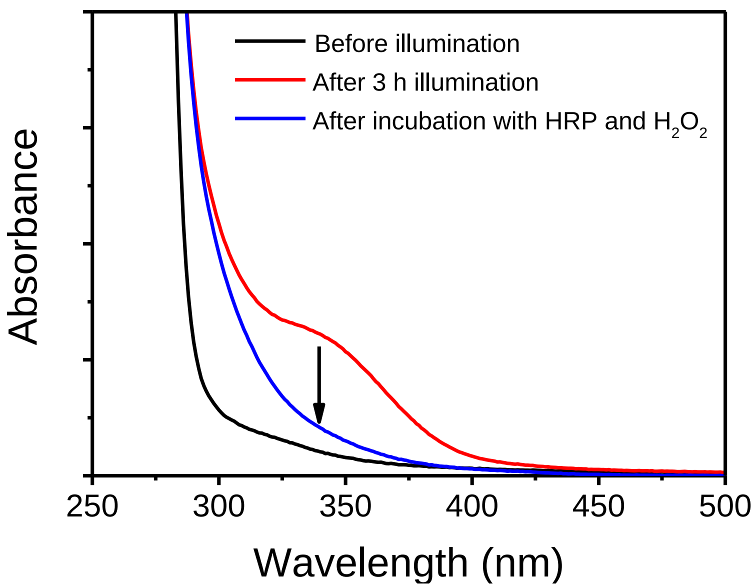

Figure S14. UV-Vis absorption spectra of the reaction system, where HRP mediated H_2O_2 reduction of regenerated NADH. After incubation with HRP and H_2O_2 for 30 min, the NADH absorbance at 340 nm decreased obviously in the HRP- catalyzed reaction, indicating that the NADH is enzymatically active (1,4-NADH). According to the absorbance change before illumination (control) and after HRP reaction, the NADH was not completely consumed in the enzymatic reaction, suggesting that the obtained NADH also consists of enzymatically inactive 1,6-NADH.

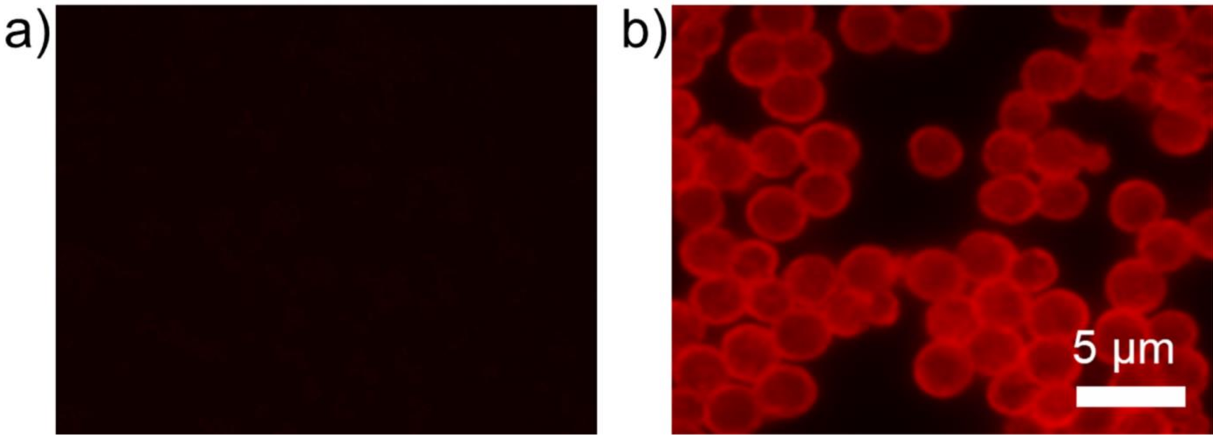

Figure S15. Fluorescent images of ZnS-Cys/Zn microspheres a) before and b) after incubation with tetrakis(4-sulfonatophenyl)porphine (TPPS) for 24 h. The excitation wavelength was 460-550 nm, and the emission was collected at ＞590 nm. The precipitates are centrifuged at 4000 rpm for 10 min and resuspended in ultrapure water before the observation. The red fluorescence, a feature of porphyrin’s emission, confirms that TPPS is encapsulated into the ZnS-Cys/Zn microspheres.

Reference

[1] F. Huang, H. Z. Zhang, J. F. Banfield, *Nano. Lett.* **2003**, *3*, 373.
